# Role of pyroptosis in diabetic cardiomyopathy: an updated review

**DOI:** 10.3389/fendo.2023.1322907

**Published:** 2024-01-05

**Authors:** Gan Wang, Tian-Yi Ma, Kang Huang, Jiang-Hua Zhong, Shi-Juan Lu, Jian-Jun Li

**Affiliations:** ^1^ Department of Cardiology, Haikou Affiliated Hospital of Central South University Xiangya School of Medicine, Haikou, Hainan, China; ^2^ State Key Laboratory of Cardiovascular Diseases, Fu Wai Hospital, National Center for Cardiovascular Diseases, Chinese Academy of Medical Sciences and Peking Union Medical College, Beijing, China

**Keywords:** pyroptosis, diabetic cardiomyopathy, NLRP3 inflammasome, inflammation, mechanism

## Abstract

Diabetic cardiomyopathy (DCM), one of the common complications of diabetes, presents as a specific cardiomyopathy with anomalies in the structure and function of the heart. With the increasing prevalence of diabetes, DCM has a high morbidity and mortality worldwide. Recent studies have found that pyroptosis, as a programmed cell death accompanied by an inflammatory response, exacerbates the growth and genesis of DCM. These studies provide a theoretical basis for exploring the potential treatment of DCM. Therefore, this review aims to summarise the possible mechanisms by which pyroptosis promotes the development of DCM as well as the relevant studies targeting pyroptosis for the possible treatment of DCM, focusing on the molecular mechanisms of NLRP3 inflammasome-mediated pyroptosis, different cellular pyroptosis pathways associated with DCM, the effects of pyroptosis occurring in different cells on DCM, and the relevant drugs targeting NLRP3 inflammasome/pyroptosis for the treatment of DCM. This review might provide a fresh perspective and foundation for the development of therapeutic agents for DCM.

## 1 Introduction

Diabetes mellitus (DM) remains a crucial public health concern. Diabetes and its complications have brought a huge medical burden to people all over the world (1, 2). There is an undeniable connection between diabetes and cardiovascular disease. Some studies have confirmed that DM can increase the likelihood of heart failure, irrespective of usual heart failure risk factors including hypertension and coronary heart disease (2). Cardiovascular complications resulting from DM are among the primary factors that contribute to mortality in patients with diabetes (1). There are currently two main types of diabetes: type 1 diabetes(T1DM), which is mainly characterized by insulin deficiency, and type 2 diabetes(T2DM), which is mainly characterized by insulin resistance (3, 4). These two main types of DM can cause microvascular and macrovascular damage, leading to a variety of diabetes-related complications such as diabetic nephropathy, diabetic cardiomyopathy (DCM), diabetic retinopathy, and so on (5).

DCM is a distinctive cardiomyopathy defined by abnormal cardiac structure and function independent of other cardiac risk factors like coronary heart disease and hypertension (6). The pathophysiological mechanisms of DCM may be partially different in different types of DM, but less is known about the differences in the pathogenesis of DCM between different types of DM. In an analysis of a multiethnic sample, Eguchi et al. found that T2DM was positively associated with increased left ventricular mass, independent of factors such as obesity and race (7). However, few studies have shown that T1DM can promote the increase of left ventricular mass, which may be related to the younger age and insulin therapy in patients with type 1 diabetes (8). Unlike T1DM, T2DM is mainly caused by hyperglycaemia due to insulin resistance and hyperinsulinemia (3, 4). Insulin signaling in cells mainly involves two related pathways: the insulin receptor substrate 1 (IRS-1) pathway and the mitogen-activated protein kinase (MAPK) pathway (9). These two pathways crosstalk to form a complex and balanced insulin signaling system. However, insulin resistance breaks this balance, making the MAPK pathway dominate, and cell growth and metabolism become imbalanced, eventually leading to cardiac fibrosis and diastolic dysfunction (6, 9).

The development of DCM can be roughly divided into two stages: the early stage is marked by left ventricular hypertrophy and diastolic dysfunction, while heart failure with systolic dysfunction characterizes the late stage (10). Various metabolic disturbances, such as hyperglycaemia and hyperinsulinaemia, are present in DM. These disturbances have been found to cause cardiac hypertrophy and diastolic dysfunction, which are the primary manifestations in most DCM patients. As a result, DCM mainly leads to heart failure with preserved ejection fraction (HFpEF) (11–13). The occurrence and development of DCM is the result of a variety of factors. Previous reviews have summarized the possible mechanisms of DCM including insulin resistance, cardiac inflammation, advanced glycation end products (AGEs), and angiotensin II (Ang II) (14, 15). Nevertheless, the precise pathogenesis of DCM remains unclear.

Cardiomyocyte death is one of the key links in the development of DCM. In the last few years, the role of various programmed cell death in DCM has received extensive attention. There are also many ways of cell death in DCM, including cell apoptosis, autophagy, cell necrosis, and ferroptosis (16, 17). Pyroptosis is a recently identified type of programmed cell death that comes with an inflammatory response. In the process of DCM, metabolic disorders such as glucose and lipids are one of the triggers of myocardial injury. Inflammation plays an essential role in it (18, 19). At the same time, many scholars believe that inflammation plays a different role in different types of heart failure. Heart failure with reduced ejection fraction (HFrEF) is mainly related to myocardial ischemia and myocarditis, while HFpEF shows a greater association with inflammation (20). Clinically, the majority of patients with DCM have predominantly HFpEF, and pyroptosis accompanied by inflammation may be an important pathogenic factor of DM and DCM. Therefore, it is imperative to investigate the related mechanisms and therapeutic interventions.

## 2 Conception of pyroptosis

Pyroptosis is one of programmed cell death (21). The term ‘ pyroptosis ‘ was first coined by Cookson and Brennan in 2001. The word consists of ‘ pyro ‘ and ‘ ptosis ‘. Pyro ‘ means fire, which means inflammation accompanied by pyroptosis, while ‘ ptosis ‘ means fall, consistent with other programmed cell death (22). Pyroptosis is mainly triggered by inflammasomes and executed by the caspase and Gasdermin families (23, 24). Furthermore, pyroptosis can manifest in diverse cell types, including the digestive system, urinary system, central nervous system, reproductive system, and cardiovascular system (25). Pyroptosis promotes the development of multiple chronic diseases (26).

## 3 Molecular mechanism of pyroptosis

**Figure 1:**
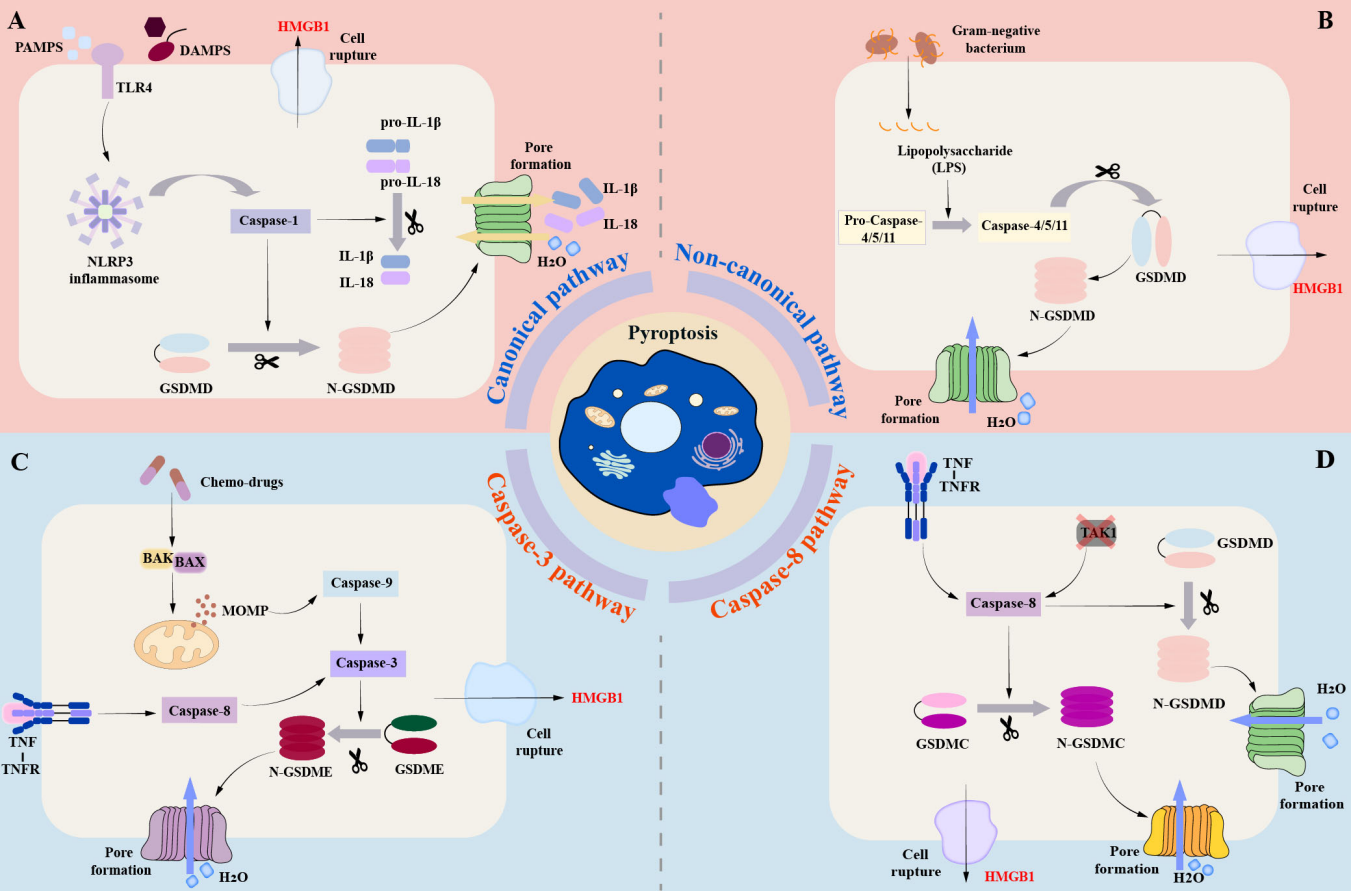
Brief molecular mechanism of Pyroptosis. **(A)** (canonical pathway): In the canonical pathway, when the host cell receptor recognizes various stimuli, it mainly includes pathogen-associated molecular patterns (PAMPs) and danger-associated molecular patterns (DAMPs), which can promote the activation of downstream pro-Caspase-1 into mature Csapase-1, and promote the assembly of inflammasome. Mature Caspase-1 can cleave GSDMD to form N-GASDMD. Subsequently, the N-terminal poreforming domain of GSDMD can non-selectively penetrate the cell membrane to form membrane pores, which further leads to cell swelling, cracking, and death. **(B)** (Non-canonical pathway): In the non-canonical pathway, LPS can directly bind to the CARD domain of Caspase-4 / 5 / 11 to achieve activation. The activated Caspase-4 / 5 / 11 can also cleave GSDMD to form N-GSDMD, thus promoting the occurrence of pyroptosis. **(C)** (Caspase-3-mediated pathway): Unlike the canonical and non-canonical pathways, activated Caspase-3 mainly mediates the formation of membrane pores by cutting GSDME and promoting the N-GSDME domain to the cell membrane, leading to the occurrence of pyroptosis. **(D)** (Caspase-8-mediated pathway): Under the stimulation of TNF-α, Caspase-8 can also specifically cleave GSDMC to produce N-GSDMC to induce pyroptosis.

The Gasdermin family and the Caspase family play an important role in pyroptosis. The Gasdermin family is predominantly found in the gastrointestinal tract and skin (27, 28). The Gasdermin family mainly consists of six proteins: GASDMA, GASDMB, GASDMC, GASDMD, GASDME, and DFNB59 (28). In addition to DFNB59, the family members exhibit two conserved domains: the N-terminal pore-forming domain and the C-terminal repression domain (28). The cleavage and release of the N domains can lead to the creation of oligomeric pores, which have a diameter of approximately 10-20 nm (29). Pyroptosis is also regulated by caspases, an evolutionarily conserved family of cysteine proteases (30). Caspases hydrolyze a variety of cellular protein substrates to coordinate cell apoptosis, and caspases are also the key players in pyroptosis (31). The molecular mechanism of pyroptosis is mainly divided into three pathways (**Figure 1**): canonical pathway, non-canonical pathway, and Caspase-3/-8-mediated pathway (32). The canonical pathway is mainly mediated by Caspase-1, while the non-canonical pathway is independent of Caspase-1, mainly mediated by Caspase-4/-5/-11 (Caspase-4/-5 in humans, Caspase-11 in mice) (28, 32).

In the canonical pathway, pattern-recognition receptors (PRRs) of the host cell are identified by recognizing a variety of stimuli, including two main categories, pathogen-associated molecular patterns (PAMPs) and danger-associated molecular patterns (DAMPs) (32, 33). Toll-like receptor 4 (TLR4), which is part of the pattern recognition receptor, is able to interact with PAMPs and DAMPs to initiate the NF-κB pathway (34). NF-κB binds to the inhibitory protein IκB and remains inactive in the cytoplasm (35). However, IKKβ phosphorylation can ubiquitinate IκBα, resulting in its dissociation from NF-κB (36, 37). Activated NF-κB is able to relocate to the nucleus, which ultimately initiates the transcription of genes associated with inflammation (38). In DCM, elevated glucose levels stimulate advanced glycation end product (AGE) synthesis, which binds with receptors for AGEs (RAGE) causing activation of the NF-κB pathway through phosphorylation of IKKβ (39). Inflammasomes are macromolecular protein complexes that play an important role in the immune system. The NLRP3 inflammasome is the most extensively studied, consisting of pyrin domain-containing NOD-like receptor protein 3 (NLRP3), CARD-containing apoptosis-associated speck-like protein (ASC), and effector protein Caspase-1 (40, 41). Typical activation of the NLRP3 inflammasome consists of two stages: initiation and subsequent activation. Initiation is mainly achieved via the nuclear factor kappa-light-chain-enhancer of activated B cells (NF-κB) pathway (34). Studies have confirmed that the activation of NLRP3 inflammasome is a key step in pyroptosis, which also plays an essential role in promoting the occurrence and development of diabetic complications such as DCM (42, 43). The activated NLRP3 inflammasome can cleave pro-Caspase to generate active Caspase-1. Activated Caspase-1 can promote the maturation of IL-1β and IL-18, in addition, it can also cleave GSDMD to form C-GSDMD and N-GSDMD. Subsequently, its N-terminal pore-forming domain oligomerizes in the membrane and binds to phosphatidic acid (PA) and phosphatidylserine (PS) to promote the formation of cell membrane pores (44). The GSDMD-mediated membrane pore is a non-selective channel that promotes the release of inflammatory substances such as IL-1β and IL-18 in cells (44–46). At the same time, extracellular fluid can enter the cell to cause cell swelling, so that the cells form bubble-like protrusions (pyroptotic bodies), eventually leading to cell membrane rupture, and finally HMGB1 and ATP flow out of the cell, causing pyroptosis (44–46).

In the non-canonical pathway mediated by Caspase-4/-5/-11, LPS can directly interact with the CARD domain of Caspase-4/-5/-11, promote the cleavage of GSDMD by active Caspase, and trigger pyroptosis (27, 32, 33, 47). Activated Caspase-4/-5/-11 could not cleave pro-IL-1β and pro-IL-18, but activated caspase-11 could cleave pannexin-1 and induce ATP release (27, 32, 33, 47). ATP binds to the P2X7 receptor and mediates potassium ion release. The efflux of ATP and potassium ions can promote the activation of NLRP3 inflammasome (41, 48). The activated NLRP3 inflammasome can promote the maturation of IL-1β and IL-18 and release them into the extracellular space through the membrane pores formed by GSDMD (27, 33, 45, 47).

Caspase-3 and Caspase-8 were previously thought to be apoptosis-related caspases, which did not stimulate GSDM to induce pyroptosis. In recent years, researchers have found that Caspase-3 and Caspase-8 can also mediate pyroptosis (32, 49, 50). Caspase-3 can induce pyroptosis by cleaving GSDME (51). Caspase-3 is mainly initiated by various chemotherapeutic drugs (52, 53). In the process of chemotherapy, chemotherapeutic drugs can initiate BAK, BAX activation, and oligomerization of the mitochondrial outer membrane, resulting in mitochondrial outer membrane permeability (MOMP) (52, 53). Subsequently, Caspase-9 and downstream Caspase-3 were activated to mediate the cleavage of GSDME, resulting in C-GSDME and N-GSDME.N-GSDME was ectopic to the plasma membrane and promoting the formation of membrane pores (54). Under the stimulation of TNF-α, Caspase-8 can specifically cleave GSDMC to produce N-GSDMC, and then induce pyroptosis (32, 49, 55). In addition, inhibition of TGF-β-activated kinase-1 (TAK1) can cause Caspase-8-dependent GSDMD cleavage during Yersinia infection, which in turn leads to pyroptosis (56). Interestingly, death receptor signaling-induced Caspase-8 can also activate Caspase-3 (57). In addition, recent studies have confirmed that GSDME and Caspase-3 can be cleaved by granzyme B at the same site (58). Granzymes can directly cleave GSDME and induce pyroptosis. At the same time, Liu et al.also found that CAR-T cells can release granzyme to activate Caspase-3 in target cells, causing extensive pyroptosis (59). Therefore, some scholars have also referred to this pathway as the granzyme-mediated pyroptosis pathway (32).

## 4 The differences between pyroptosis and apoptosis

Pyroptosis and apoptosis are both programmed cell death, and there are some similarities between them. For example, both of them rely on Caspases, which damage DNA and cell membrane blebbing (29, 32, 60). However, Regardless of morphology or mechanism, pyroptosis is different from apoptosis (**Table 1**). Compared with apoptosis, pyroptosis is significantly different in that pyroptosis can promote the occurrence of inflammation, which can lead to cell swelling and incomplete cell membranes (29, 32). In addition, although both of them can cause DNA damage, unlike apoptosis, the nucleus remains intact during pyroptosis, and the nucleus is broken when apoptosis occurs (61, 62). The plasma membrane undergoes diverse morphological alterations during these two processes. During apoptosis, apoptotic bodies can form, while pyroptotic bodies may develop during pyroptosis (29). Apoptotic bodies are subcellular structures that consist of intracellular material, including DNA, organelles, and nuclear debris, and their formation is dependent on membrane blebbing (63, 64). Pyroptotic bodies are a new cellular morphological structure discovered by observing cells undergoing pyroptosis through Time-lapse electron microscopy (29). The nature of pyroptotic bodies remains unclear, but it is interesting to note that pyroptotic bodies share a resemblance to apoptotic bodies, with diameters of about 1-5 μm (45). Furthermore, pyroptosis undermines the integrity of the plasma membrane. Cell death was previously usually classified as apoptosis and necrosis (65, 66). Apoptosis is an active, programmed, non-inflammatory form of cell death. Whereas necrosis is a passive, accidental, non-programmed, with inflammatory response (65, 66). Subsequently, the researchers uncovered another form of cell death that has the characteristics of necrosis but can be highly regulated, called regulated necrosis (67, 68). Both necroptosis, also referred to as programmed necrosis, and pyroptosis are forms of regulated necrosis, characterized by the disruption of cytoplasmic membrane integrity and cellular content leakage (67, 69). There are numerous similarities between necroptosis and pyroptosis, both of which have the potential to initiate inflammation (70). Furthermore, studies conducted on animals have indicated that necroptosis is significant in the occurrence of myocardial infarction, renal ischemia, and ischemia-reperfusion in stroke (71–73). RAW-asc cells have the ability to undergo necroptosis and pyroptosis when exposed to various stimuli. Through electron microscopy observation, Chen et al. noted that necroptotic cells become rounded and eventually rupture explosively, while pyroptosis produces flat pyroptotic bodies before cell rupture occurs. This research also affirms that pyroptosis, in contrast to MLKL channel-mediated necroptosis, requires the creation of non-selective pores by GSDMD-N to enable cell lysis (45).

**Table 1:** The differences between pyroptosis and apoptosis.

Characteristics	Pyroptosis	Apoptosis
membrane rupture	YES	NO
Cell swelling;	YES	NO
Nucleus intact	YES	NO
Inflammation	YES	NO
Pore formation	YES	NO
Apoptotic bodies	NO	YES
pyroptotic bodies	YES	NO
Caspase-1	YES	NO
Caspase-2	NO	YES
Caspase-4	YES	NO
Caspase-5	YES	NO
Caspase-7	NO	YES
Caspase-10	NO	YES
Caspase-11	YES	NO
Caspase-12	YES	NO

The caspase family functions critically in pyroptosis as well as apoptosis. Caspases, when activated, can lead to programmed cell death. Additionally, they can also determine the type of cell death that takes place (30, 31, 74, 75). According to their functions, mammalian Caspase can be classified into two types: Apoptotic caspases and Inflammatory caspases (75). Apoptotic caspases encompass Caspase-2, -7, and -10, while inflammatory caspases mainly include Caspase-1, -4, -5, -11and-12 (30, 75, 76). Although the Caspases involved in apoptosis and pyroptosis are not all the same, Caspase-3,-6,-8,-9 have now been shown to play a role in both apoptosis and pyroptosis (32). Inflammatory caspases can accomplish pyroptosis by cleaving different gasdermin. In contrast to inflammatory caspases, apoptotic caspases primarily execute a type of programmed cell death termed apoptosis, which is immunologically silent (77). Depending on their role in the process of cell death, caspases can be classified as either initiation proteins, including caspase-2, -8, -9, and -10, or effector proteases, namely caspase-3, -6 and -7 (77). Notably, most of the caspases that induce pyroptosis can also induce apoptosis in the absence of the corresponding GSDM proteins, implying that cleavage of GSDM by inflammatory caspases can convert apoptosis into pyroptosis (78). Meanwhile, specific cleavage of GSDM by inflammatory caspases is necessary for pyroptosis (74).

## 5 Mechanisms of pyroptosis-triggered DCM

**Figure 2:**
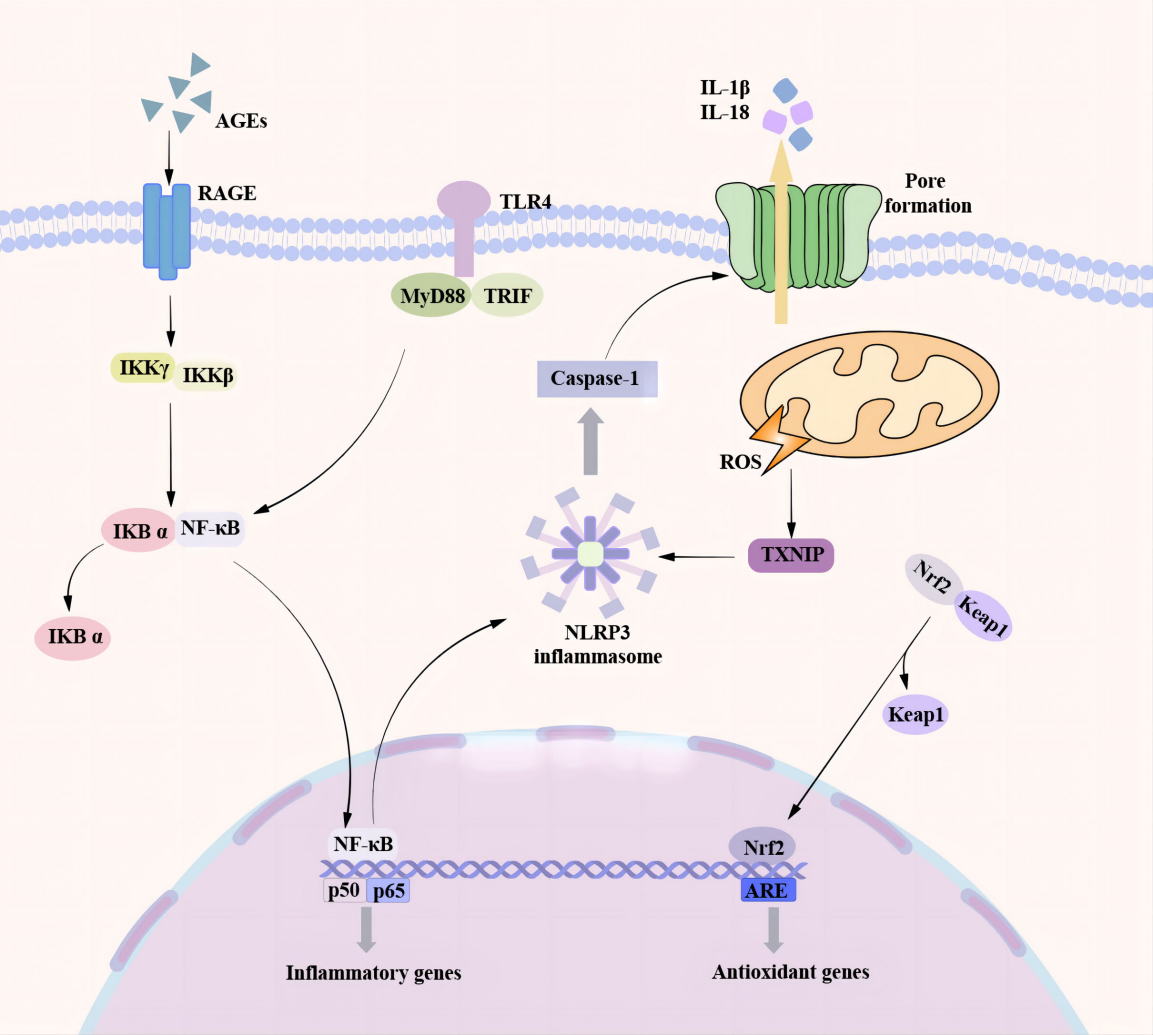
Pyroptosis pathway associated with DCM. There are many pathways involved in the process of pyroptosis promoting the occurrence and development of DCM. In the NF-κB / NLRP3 signaling pathway, the TLR4 receptor senses various stimulating factors, separates the NF-κB in the cytoplasm from the inhibitory protein IκB, and transfers to the nucleus, resulting in an increase in the expression of NLRP3 and the activation of NLRP3 inflammasome. A high glucose environment can induce the production of a large number of ROS. TXNIP is a ROS-dependent NLRP3 inflammasome activation regulator. A large amount of ROS can promote the binding of TXNIP to NLRP3 and trigger the activation of NLRP3 inflammasome. As a transcription factor, Nrf2 can regulate cardiac homeostasis by controlling various antioxidant genes to inhibit oxidative stress. Nrf2 is transferred to the nucleus under the action of ROS and oxidative stress and binds to the promoter region of the antioxidant response element (ARE) to promote the production of antioxidant enzymes and protect cardiomyocytes. At the same time, Nrf2 can also inhibit NF-κB to reduce the formation of NLRP3 inflammasome and inhibit pyroptosis.

**Figure 3:**
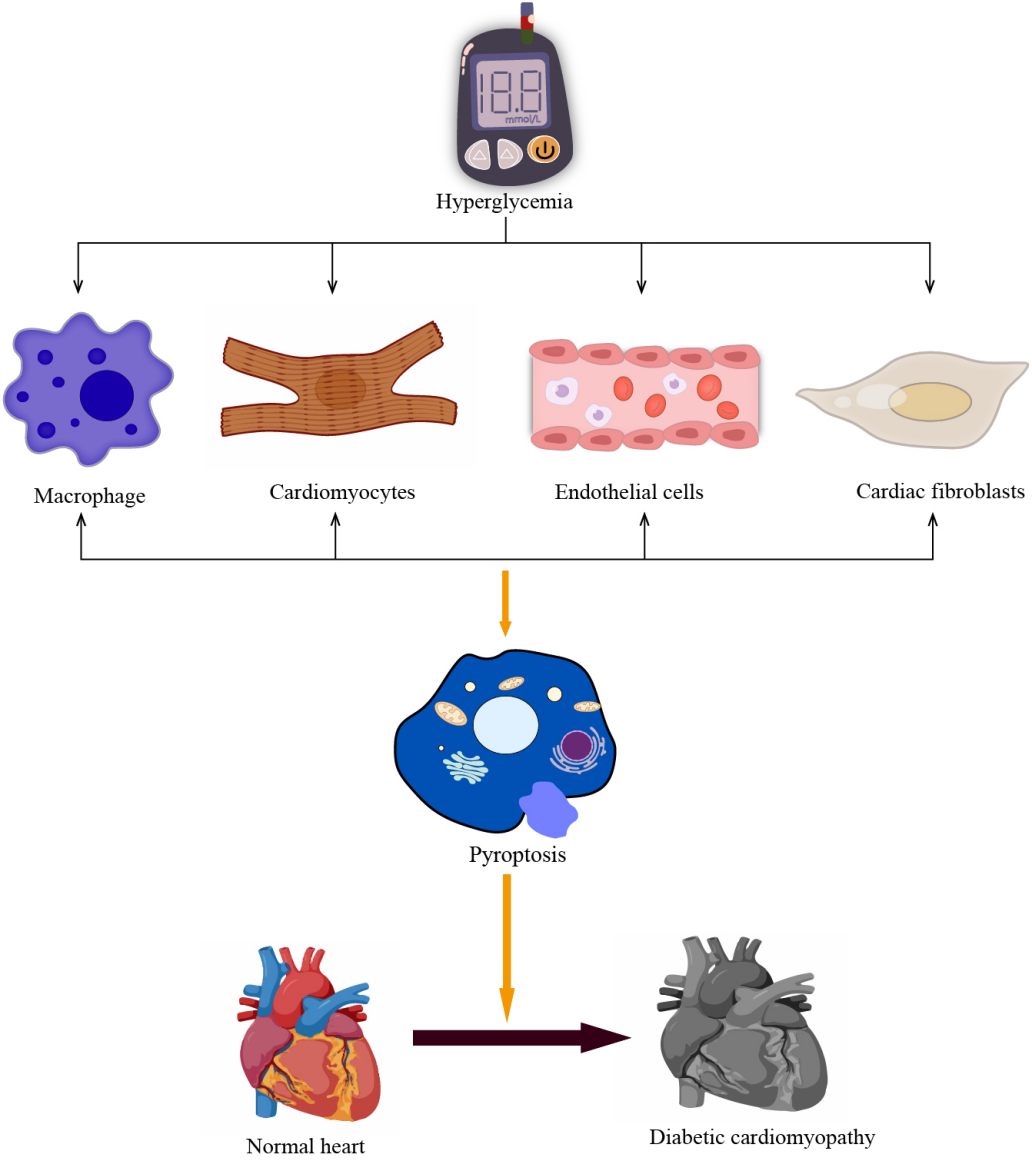
Pyroptosis in different cells promotes the development of DCM. The prolonged hyperglycemia causes pyroptosis of non-myocardial cells such as cardiomyocytes and macrophages, vascular endothelial cells, and cardiac fibroblasts, promoting poor cardiac remodeling and accelerating the development of DCM.

The development of DCM is a complex process driven by multiple factors. Chronic inflammation is one of the key drivers of DCM development. Chronic inflammation can lead to cellular death by triggering the activation of inflammatory vesicles such as NLRP3 (79, 80). Pyroptosis can promote the development of DCM through multiple pathways (**Figure 2**), and both myocardial cells and non-myocardial cells can undergo pyroptosis to accelerate the development of DCM (**Figure 3**) (25). Understanding the effects of the different pathways of pyroptosis and the cell types that undergo cellular pyroptosis on DCM may provide possible therapeutic targets for DCM.

### 5.1 NLRP3 regulatory pathways associated with DCM

#### 5.1.1 NF-κB/NLRP3 inflammasome pathway

The NF-κB pathway promotes the activation of NLRP3 inflammasome, which are involved in multiple chronic inflammatory diseases such as Ulcerative Colitis, lupus nephritis, rheumatoid arthritis, and so on (81–83). In recent years, NF-κB-related signaling pathways have been recognized as crucial in the pathophysiology of DCM. Luo et al. observed severe metabolic disturbances in diabetic rats and increased expression of NLRP3, ASC, caspase-1, and IL-1β in a rat model of type 2 diabetes induced by a high-fat (HF) diet and low-dose streptozotocin (STZ). Furthermore, it was shown that NF-κB is involved in the activation of NLRP3 inflammasome in high glucose-induced H9C2 cells (84). Another study demonstrated that the use of tilianin and syringin in this model not only ameliorated metabolic disorders and improved cardiac function in diabetic rats but also significantly increased the expression of TLR4, NF-Kb, NLRP3, IL-1β (85).In the STZ-induced C57BL/6 mouse model, it was observed that DCM was prevented in STZ-induced diabetic mice by inhibiting NF-κB and NLRP3 (86). High glucose (HG) enhances the expression of IL-1β, NLRP3, caspase-1, and ASC along with TLR4 protein levels in HUVECs. These effects were reversed upon TLR4 silencing (87). Besides, the study revealed that in H9C2 cells, high glucose led to an increased expression of NLRP3 inflammatory vesicles, TLR4, and NF-κB. However, the utilization of exogenous H2S effectively inhibited the activation of the TLR4/NF-Kb/NLRP3 pathway (88). All the experiments mentioned indicate the crucial involvement of TLR4/NF-κB/NLRP3 inflammasome pathway in the onset and progression of DCM. Targeting a specific link in this pathway can offer a new therapeutic approach for DCM.

#### 5.1.2 Reactive oxygen species/thioredoxin interacting protein/NLRP3 inflammasome pathway

ROS is a by-product produced by specific enzymes during mitochondrial respiration or metabolism (89). ROS has two sides, it has a beneficial effect while also causing damage to the body (89). Long-term adverse environments such as hyperglycaemia, hyperlipidemia, and chronic inflammation can lead to the up-regulation of ROS-producing enzyme activities, resulting in a large amount of ROS and inducing oxidative stress (89, 90). It has been shown that HG causes a significant increase in ROS production in H9C2 cardiomyocytes, leading to the activation of NLRP3 inflammatory complexes via cytochrome C-dependent pathways (91). TXNIP serves as a regulator of NLRP3 inflammasome activation dependent on ROS. Researchers have shown that ROS promotes the binding of TXNIP to NLRP3, leading to the activation of the NLRP3 inflammasome (92–94). Suppression of ROS significantly diminished the expression of TXNIP, NLRP3 inflammatory vesicles, and IL-1β in H9C2 cells induced with high glucose. Meanwhile, suppression of TXNIP expression through the use of TXNIP-siRNA plasmid decreased downstream caspase-1 and IL-1β activation. Moreover, Silencing NLRP3 attenuated the expression of caspase-1 and IL-1β, while alleviating left ventricular dysfunction and reversing myocardial remodeling in diabetic rats on the HF diet and in low-dose STZ-induced diabetic rats (95). These indicate that ROS and the resulting oxidative stress can cause negative changes in the structure of the heart and hasten the progression of DCM (89, 93). Furthermore, in a rat INS-1 pancreatic β-cell-related study, Liu et al. found that HG significantly increased the expression of TXNIP, NLRP3, and caspase-1 in the cells and that TXNIP showed a dose-dependent relationship with glucose concentration and that TXNIP expression increased with increasing glucose concentration (96). These all suggest that the ROS/TXNIP/NLRP3 inflammasome pathway plays an important role in DM and may accelerate the progression of DCM.

#### 5.1.3 Nrf2-related pathways

Nuclear factor-erythroid-2-related factor 2 (Nrf2) is a transcription factor that is expressed in a variety of tissues and organs. Studies have demonstrated that the Nrf2 signaling pathway is closely related to various cardiac diseases, which can regulate cardiac homeostasis by inhibiting oxidative stress by controlling various antioxidant genes (97). Under physiological conditions, Nrf2 binds to and is present in the cytoplasm with its repressor Kelch-like epichlorohydrin-associated protein 1 (Keap1) (97, 98). However, large amounts of ROS and oxidative stress dissociate Nrf2 from Keap1, causing Nrf2 to transfer to the nucleus to bind to the promoter region of the antioxidant response element (ARE), which activates antioxidant genes and promotes antioxidant enzyme production (97, 98). These antioxidant enzymes have anti-inflammatory and antioxidant effects, protecting cardiomyocytes from myocardial damage caused by diabetes and high glucose oxidation (98). A study showed that Nrf2 upregulated the antioxidant protein HO-1 expression in STZ-induced SD rats and HG-cultured H9C2 cells, suppressed cardiomyocyte pyroptosis and impeded the progression of DCM (99). In contrast, blocking the Nrf2 pathway with ML385 did not decrease the expression of proteins associated with cellular pyroptosis (NLRP3, caspase-1, etc.). Furthermore, this study discovered that Nrf2 activation impedes NF-κB, leading to a decrease in the production of NLRP3 inflammasomes and the inhibition of pyroptosis, ultimately leading to cardioprotection (99). Li et al. observed similar results in C57BL/6 mice, where the use of Luteolin increased Nrf2 expression, attenuated cardiac oxidative stress, and provided cardioprotection (100). Another experiment using the Nrf2 knockout vector showed that cardiomyocytes were highly susceptible to high glucose-induced injury. The expression of NLRP3, caspase-1, IL-18, and IL-1β was also significantly increased. Meanwhile, DM rats treated with ML385 to suppress Nrf2 showed more severe myocardial fibrosis and cardiac enlargement (101). All of these studies provide valuable evidence that the Nrf2-related pathway may serve as a future target for treating DCM.

### 5.2 Effect of cardiomyocyte pyroptosis on DCM

Pyroptosis can occur in various cells of the heart, including cardiomyocytes, macrophages, fibroblasts, and endothelial cells. Cardiomyocytes are the main cells that make up the heart. Cardiomyocytes enable the heart to perform systolic and diastolic functions and help the heart pump blood throughout the body (102). The death of cardiomyocytes, whether acute or chronic, can cause irreversible damage to the heart. The activation of NLRP3 inflammasome plays an important role in the process of cardiomyocyte pyroptosis promoting cardiac dysfunction (43, 103, 104). When myocardial ischemia occurs, the potassium efflux in cardiomyocytes increases, thereby up-regulating the expression of NIMA-associated kinase 7 (NEK7) and promoting the activation of NLRP3 inflammasome (105–108). The activation of NLRP3 inflammasome in cardiomyocytes under pressure overload mainly depends on the regulation of NF-κB by calcium/calmodulin-dependent kinase II (CaMKII) (109–111). Hyperglycaemia promotes high ROS production, which binds TXNIP to NLRP3, thereby promoting the activation of NLRP3 inflammasome (43). Moreover, a high glucose environment can promote the pyroptosis of various cells in the heart, thereby accelerating the development of DCM (43). The death of cardiomyocytes plays an important role in the progression of DCM (80). New evidence also confirms that cardiomyocyte pyroptosis induced by NLRP3 inflammasome is a critical step in the progression of DCM (43, 112).

An experiment demonstrated that modulation of the miR-34b-3p/AHR axis in high glucose-induced HL-1 cells inhibited the activation of NLRP3 inflammasome and attenuated NLRP3-mediated pyroptosis in HL-1 cells. Furthermore, the hindrance of pyroptosis resulted in enhanced cardiac function and a reduction in cardiac hypertrophy among DCM mice (113). The expression of the NLRP3 inflammasome has been found to increase when PNRCMs are exposed to HG. This leads to pyroptosis in these cells (114). Likewise, within cardiomyocytes, such as those induced by HGin AC-16 and H9C2 cell lines, as well as primary cardiomyocytes separated from neonatal C57BL/6 mice, there was an increase in the expression of specific focal death-associated proteins, including caspase-1, NLRP3, and other proteins (115, 116). The conducted *in vivo* and *in vitro* experiments exhibit that suppressing cardiomyocyte pyroptosis ameliorates cardiac function and adverse remodeling besides delaying DCM progression. Furthermore, these outcomes highlight pyroptosis as a plausible therapeutic target for DCM.

### 5.3 Impact of non-cardiomyocyte pyroptosis on DCM

#### 5.3.1 Macrophages

Macrophage pyroptosis also plays an important role in complications caused by hyperglycaemia (117). Macrophages constitute a vital aspect of the immune system, performing functions such as identification, phagocytosis, secretion, regulation of immunity, and maintenance of body homeostasis (118, 119). A study found that in diabetic mouse models, persistent hyperglycaemia can lead to macrophage dysfunction in which activation of NLRP3 inflammasome plays an important role. Partial macrophage dysfunction induced by hyperglycaemia can be restored by inhibiting the activation of cathepsin B and NLRP3 inflammasome (117). Simultaneously, researchers discovered that the activation of NLRP3 inflammasome in M1 macrophages can exacerbate cardiac dysfunction and encourage myocardial morphological changes in a post-stroke diabetic mouse model. The implementation of CY-09 to inhibit NLRP3 inflammasome has been shown to ameliorate cardiac function in diabetic mice (119). These findings suggest that the activation of NLRP3 inflammasome and pyroptosis in macrophages may contribute to the development of DCM.

#### 5.3.2 Fibroblasts

DCM may manifest cardiac interstitial fibrosis in addition to cardiomyocyte hypertrophy. Cardiac fibroblasts are effector cells in the process of myocardial fibrosis (120). When the heart suffers various injuries, cardiac fibroblasts can repair the heart by promoting the formation of collagen and extracellular matrix. However, when the damage is excessive, this repair matrix causes cardiac fibrosis, resulting in a decrease in cardiac compliance. In the diabetic state, metabolites such as glycosylation end products (AGEs) promote fibroblast differentiation into myofibroblasts (120, 121). Pyroptosis also plays an important role in the process of cardiac fibrosis. Ren et al. discovered that HG caused an increase in caspase-1, IL-1β mRNA, and protein expression in fibroblasts, as well as a similar trend in GSDMD protein expression. However, the administration of ranolazine led to a reduction in cardiac fibrosis in diabetic rats by inhibiting fibroblast pyroptosis and decreasing collagen deposition through the regulation of miR-135b (122). Another study also indicated that the expression of NLRP3, IL-1β, and GSDMD-N increased in fibroblasts following inducement by high glucose. Conversely, the inhibition of inflammation and pyroptosis in fibroblasts improved cardiac function and reduced fibrosis in diabetic C57BL/6 mice (123). These studies all confirm that fibroblasts play an important role in the progression of DCM and that modulation of fibroblast pyroptosis may alleviate cardiac fibrosis and delay the onset of DCM.

#### 5.3.3 Vascular endothelial cells

Vascular endothelial cell dysfunction is also the key pathological basis for the occurrence and development of DCM (124). Vascular endothelial cells form a semi-permeable barrier between circulating blood and the extravascular matrix, and this endothelial barrier regulates cellular connectivity to maintain homeostasis in the body (125). During the development of DCM, activated NLRP3 inflammasome induces cellular pyroptosis releasing large quantities of pro-inflammatory factors IL-1β and IL-18. These pro-inflammatory factors subsequently bind to cell-surface receptors and enhance the expression of adhesion and chemokine in the endothelium, while increasing leukocyte adhesion and extravasation. This ultimately leads to the disruption of intercellular junctions, endothelial barrier dysfunction, and increased vascular permeability, which also facilitates the infiltration of pro-inflammatory cells and pro-inflammatory factors and accelerates adverse cardiac remodeling (124, 125). Increased NLRP3, caspase-1, and other cell death-related proteins were observed in HUVEC cells exposed to HG (126). A further study has verified that NLRP3 inflammasome-induced pyroptosis, can harm vascular endothelial cells and reduce the density of cardiac microvessels, resulting in negative cardiac restructuring (127).

## 6 Potential therapies of pyroptosis/NLRP3 in DCM

The pathogenesis of DCM is very complex. Although its mechanism has been continuously improved for decades, there is still no effective treatment for DCM, which also makes the morbidity and mortality of DCM still high. As many researchers have found that pyroptosis plays an important role in DCM, targeted inhibition of pyroptosis and NLRP3 inflammasome may provide a good direction for the treatment of DCM. At present, these substances mainly include hypoglycemic drugs, phytochemicals, NLRP3 inflammasome, pyroptosis inhibitors, non-coding RNA, and so on (**Table 2**).

**Table 2:** Therapies and mechanisms targeting NLRP3 inflammasome/pyroptosis.

Intervention strategies	Targeted therapies	Models	Mechanisms
**Hypoglycaemic drugs**	Empagliflozin	Spontaneous type 2 diabetic db/db mice	Reducing the activation of NLRP3 inflammasome and inhibiting pyroptosis; Regulating the activity of sGC-cGMP-PKG pathway to inhibit oxidative stress;
	Dapagliflozin	diabetic BTBR ob/ob mice	Inhibition of NLRP3 inflammasome activation via AMPK / mTOR axis; Combined with ticagrelor to inhibit the interaction of NLRP3 inflammasome through an AMPK-mTOR interplay;
	Metformin	streptozotocin-induced mice; High glucose-treated primary cardiomyocytes	Activate AMPK; Inhibition of mTOR pathway; Activating the PK2/PKR Pathway;
**Inhibitor compounds**	MCC950	High glucose-treated primary cardiomyocytes	Binding to the Waller B site in the NACHT domain of NLRP3;
	CY-09	diabetic db/db mice	Binding to the Waller A site in the NACHT domain of NLRP3;
**Non-coding RNA**	miR-30d	streptozotocin (STZ)-induced diabetic rats; High-glucose-treated cardiomyocytes;	Regulating cardiomyocyte pyroptosis by directly targeting foxo3a
	LncRNA KCNQ1OT1	STZ-induced mice; High-glucose-treated AC16 cells and primary cardiomyocytes;	Targeting miR-214-3p and caspase-1
	CircRNA DICAR	DICAR+/− and DICARTg mic	DICAR-VCP-Med12 degradation
**Phytochemicals**	Quercetin	STZ -induced diabetic rats; High-glucose-treated H9C2 cells;	Promoting nuclear Nrf2 nuclear translocation.
	Pomegranate peel extract	STZ-induced diabetic rats;	Inhibition of NLRP3 / caspase-1 / IL-1β signaling pathway; Down-regulation of lncRNA-MALAT1;

### 6.1 Hypoglycaemic drugs

Sodium-glucose co-transporter 2 inhibitor (SGLT2i) is one of the commonly used hypoglycemic drugs. SGLT2i plays a hypoglycemic role mainly by increasing urinary glucose excretion. In addition to lowering blood glucose, SGLT2i also has an extraordinary cardioprotective effect, which can reduce cardiovascular mortality and heart failure hospitalization rates in patients with type 2 diabetes (128). SGLT2i plays a role in delaying the progression of DCM by regulating metabolism, improving mitochondrial function, inhibiting oxidative stress, and reducing programmed cell death (129).

Empagliflozin is one of the typical SGLT2i, which has been widely used in patients with diabetes (130). A study showed that empagliflozin reduced the activation of NLRP3 inflammasome and attenuated pyroptosis caused by NLRP3 inflammasome in diabetic mice. In addition, this study also found that empagliflozin can inhibit oxidative stress by affecting the activity of the sGC-cGMP-PKG pathway, thus exerting cardioprotective effects (130). Dapagliflozin can inhibit SGLT2 to reduce NLRP3 inflammasome activation and delay the progression of DCM in type 2 diabetic mice. At the same time, it has a better inhibitory effect when combined with DPP-4 inhibitor Saxagliptin (131). It has also been found that ticagrelor, a P2Y12 receptor antagonist, acts synergistically with dagliflozin to slow the progression of DCM by attenuating the activation of NLRP3 inflammatory vesicles via the AMPK/mTOR axis (132).

Metformin is a kind of biguanide drug derived from herbaceous plants. It is the first-line treatment of diabetes. It can protect the heart in a variety of ways (133). Metformin was found to improve DCM by down-regulating the expression of NLRP3, Caspase-1, and IL-1β through AMP-activated protein kinase (AMPK) in cardiomyocytes treated with high glucose and diabetic mouse models (134). Studies have also shown that metformin can reduce the adverse effects of hyperglycaemia on streptozotocin-induced diabetic mice through the PK2/PKR pathway (135). Metformin can also produce incredible results when co-administered with other drugs. Ye et al. found that metformin significantly suppressed oxidative stress and inflammation when co-administered with the lipid-lowering drug atorvastatin, providing better protection against DCM (131). Metformin combined with hydrogen, Cocoa-Carob Blend, and Dendrobium Mixture can also show more effective cardioprotective effects (136–138).

In addition, certain hpoglycaemic drugs have demonstrated the ability to impede cellular pyroptosis in non-diabetic conditions. This implies that glucose-lowering drugs may potentially enhance DCM by inhibiting cellular pyroptosis. Zhao et al. induced HUVEC cells with oxidized LDL and observed that SGLT2i decreased cellular pyroptosis while enhancing endothelial cell dysfunction (139). In the case of ischemia/reperfusion injury, metformin inhibits NLRP3 inflammasome activation and exerts cardioprotective effects through the AMPK pathway (140).

### 6.2 Inhibiting compounds

NLRP3 inflammasome plays an important role in pyroptosis promoting the occurrence and development of DCM (42, 43). Inhibition of NLRP3 inflammasome can greatly inhibit pyroptosis and delay the progression of DCM. MCC950 is a small molecule that can effectively inhibit NLRP3 inflammasome (141). MCC950 binds to the Waller B site in the NACHT domain of the NLRP3 inflammasome, thereby blocking ATP hydrolysis and inhibiting NLRP3 inflammasome formation (142, 143). In high glucose-induced cardiomyocytes, the use of MCC950 can inhibit the expression of NLRP3, down-regulate the expression of pyroptosis-related proteins, and alleviate high glucose-induced LDH leakage (114). In a study in streptozotocin-induced diabetic mice, MCC950 was also found to delay the development of various complications associated with diabetes, including diabetic retinopathy, diabetes-associated atherosclerosis, and diabetic encephalopathy, by inhibiting the NLRP3 inflammasome and its downstream inflammatory response (144–146). The effect of MCC950 on DCM is still insufficient, but the above experimental results are undoubtedly encouraging. The exact effect of MCC950 on DCM and its related mechanisms still needs to be improved by researchers.

CY-09 is a compound that can effectively inhibit NLRP3 by directly binding to the ATP-binding motif of the NACHT domain of NLRP3 and inhibiting NLRP3 ATPase activity, thereby inhibiting the assembly and activation of the NLRP3 inflammasome (147). Jiang et al.also studied the mechanism of CY-09 inhibiting NLRP3 ATPase activity. The NLRP3 NACHT domain contains two sequences that are important for ATPase activity. The Walker A motif is an important motif for ATP binding, while the Walker B motif is essential for ATPase activity (148). Unlike MCC950, CY-09 binds to the Waller A site in NLRP3, thereby blocking the binding of NLRP3 to ATP, thereby blocking ATPase activity (147). CY-09 significantly ameliorates metabolic disorders in a diabetic mouse model (147), and can also alleviate inflammation, oxidative stress, and fibrosis in diabetic mice by selectively inhibiting NLRP3 inflammasome, thereby improving renal damage in diabetic nephropathy (149). Furthermore, CY-09 also alleviated insulin resistance and hepatocyte steatosis in diabetic mice (150). In terms of cardiac protection, CY-09 can inhibit the activation of NLRP3 inflammasome in M1 polarized macrophages and improve cardiac dysfunction after ischemic stroke (119). The above experiments show that CY-09 may also be used to delay DCM, but there are few studies on the relationship between CY-09 and DCM.

### 6.3 Non-coding RNA

In the process of pyroptosis accelerating the occurrence and development of DCM, non-coding RNA also plays an important regulatory role. Non-coding RNA (ncRNA) includes many types, including microRNA (miRNA), long non-coding RNA (lncRNA), and circular RNA (circRNA) (151). Among them, miRNA is the most studied type of ncRNA (151). MiRNA is a class of endogenous small non-coding RNA molecules with a length of about 20 nucleotides. It can play a role in biological processes such as cell proliferation, differentiation, and apoptosis by inhibiting or activating gene expression (152). MiRNAs can be stably expressed in different body fluids, and the level of this miRNA in the blood can also change at different stages of the development of DCM (152). Therefore, miRNA may be a potential biomarker for DCM (152, 153). MiR-21, miR-30d, miR-223, and other miRNAs were found to be up-regulated in DCM, whereas miR-1, miR-9, miR-150, and other miRNAs were found to be down-regulated in diabetic conditions (153, 154). Recent studies have found that some miRNAs can slow down the development of DCM by regulating pyroptosis. *In vivo* and *in vitro* experiments confirmed that the expression of miRNA-30d in the diabetic group was significantly increased, and its increased expression up-regulated the expression of pro-inflammatory factors such as Caspase-1 and IL-1βand directly inhibited the expression of foxo3a and its downstream proteins, which promoted the pyroptosis of cardiomyocytes in DCM. However, these effects were reversed after the knockdown of miR-30d (155). Xu et al. also found that miR-223 is highly expressed in H9C2 cardiomyocytes induced by high glucose, and the use of miR-223 inhibitors can attenuate the activation of NLRP3 inflammasome and alleviate myocardial fibrosis, thereby delaying the development of DCM and protecting the heart (154). In the diabetic mouse model induced by high glucose and high fat, Deng et al.also found that miR-223 can regulate the expression of NLRP3 to reduce damage to endothelial cells (156). Meanwhile, it also shows that in the development of DCM, in addition to the myocardial cells themselves, endothelial cell death also plays an important role in promoting it.

In recent years, lncRNAs and circRNAs have also been found to play a regulatory role in DCM. LncRNAs refer to transcription RNA molecules with a length of more than 200 nucleotides, but they do not have protein-coding ability (157). LncRNA KCNQ1OT1 expression is increased in diabetic patients, high glucose-induced cardiomyocytes, and diabetic mouse models. Silencing KCNQ1OT1 inhibits pyroptosis by targeting miR-214-3p and Caspase-1, and also ameliorates abnormal cytoskeleton structure and calcium overload, improving cardiac structure and function (116). CircRNAs are joint regulators in various diseases. CircRNA DICAR has been shown to alleviate DCM, and knockout of DICAR can enhance pyroptosis in DCM (158).

### 6.4 Phytochemicals

In recent years, some substances extracted from herbs have received extensive attention in academia due to their anti-inflammatory effects. Flavonoids are the most abundant phytochemicals in plants, which can alleviate DCM by reducing myocardial oxidative stress and inflammation, so they have also been widely studied (159). Quercetin is a natural flavonoid. Zhang et al.found that quercetin increased the expression of antioxidant proteins such as HO-1 through the Nrf2 pathway and inhibited myocardial pyroptosis (99). Pomegranate peel extract was found to improve cardiac hypertrophy and myocardial fibrosis in diabetic rat models. The study also found that pomegranate peel extract may protect DCM by inhibiting the NLRP3/caspase-1/IL-1β signaling pathway and down-regulating the expression of lncRNA-MALAT1 (160).

Colchicine is a tricyclic alkaloid that is mainly used to treat inflammatory diseases such as gout. In cardiovascular diseases, colchicine is used in acute and recurrent pericarditis, coronary syndrome, atrial fibrillation, and heart failure (161). Colchicine can inhibit the activation of NLRP3 inflammasome by inhibiting the P2X7 receptor and blocking potassium efflux. Moreover, colchicine can also inhibit microtubule synthesis, promote microtubule degradation, and inhibit the assembly of NLRP3 inflammasome (162). Given the important role of colchicine in inhibiting the activation of NLRP3 inflammasome, it may have the potential to treat DCM. However, there are few studies on the role of colchicine in DCM.

## 7 Conclusion

DCM has been recognized for decades, and a large number of studies have been carried out to explore its potential pathophysiological mechanisms. However, the incidence of DCM is still increasing with the increasing prevalence of diabetes. The pathogenesis of DCM has been refined through continuous exploration by researchers, and several new drugs have been shown to have beneficial effects *in vivo* and *in vitro* models. Pyroptosis, as a programmed cell death with inflammation, plays an important role in the occurrence and development of DCM. Understanding the different pyroptosis pathways associated with DCM and the effects of pyroptosis of different cells on DCM will help us to find new therapeutic targets. Some researchers have also found that the intervention of pyroptosis by hypoglycemic drugs and inhibitors targeting NLRP3 inflammasome can delay the progression of DCM. However, the exact mechanism between DCM and pyroptosis has not yet been clarified, and it still needs further exploration by researchers.

## Author contributions

GW: Writing – original draft, Writing – review & editing. TM: Writing – review & editing. KH: Writing – review & editing. JZ: Writing – review & editing. SL: Writing – review & editing. JL: Writing – review & editing.
